# Coagulation assay results at birth in preterm infants: A cohort study highlighting the relevance of local reference values for interpretation

**DOI:** 10.1111/vox.13766

**Published:** 2024-11-18

**Authors:** Nina Houben, Suzanne Fustolo‐Gunnink, Camila Caram‐Deelder, Remco Visser, Madeleen Bosma, Karin Fijnvandraat, Jeroen Eikenboom, Johanna van der Bom, Enrico Lopriore

**Affiliations:** ^1^ Division of Neonatology, Willem‐Alexander Children's Hospital Leiden University Medical Center Leiden the Netherlands; ^2^ Sanquin Research Sanquin Blood Supply Foundation Amsterdam the Netherlands; ^3^ Department of Clinical Epidemiology Leiden University Medical Center Leiden the Netherlands; ^4^ Department of Clinical Chemistry and Laboratory Medicine Leiden University Medical Center Leiden the Netherlands; ^5^ Department of Paediatric Haematology, Emma Children's Hospital, Amsterdam UMC University of Amsterdam Amsterdam the Netherlands; ^6^ Department of Molecular Cellular Haemostasis Sanquin Research, Sanquin Blood Supply Foundation Amsterdam the Netherlands; ^7^ Department of Internal Medicine, Division of Thrombosis and Haemostasis Leiden University Medical Center Leiden the Netherlands

**Keywords:** activated partial thromboplastin time (APTT), coagulation assay, neonatal intensive care unit (NICU), plasma transfusion, preterm infant, prothrombin time (PT)

## Abstract

**Background and Objectives:**

Routine coagulation screens at birth are still standard in some European neonatal intensive care units (NICUs), although interpretation of these results is complex in preterm infants. It is unclear to what extent local coagulation assay results agree with published reference ranges when using different analysers and reagents. We aimed to assess coagulation assay results on day 1 of life in very preterm infants admitted to the NICU.

**Materials and Methods:**

We included all preterm infants born below 32 weeks gestational age (GA) admitted to the Leiden University Medical Center between 2004 and 2020 in whom coagulation assays (prothrombin time [PT] and activated partial thromboplastin time [APTT]) were obtained during the first 24 h of life. Infants either diagnosed with major intraventricular haemorrhage or who received plasma transfusion before coagulation assay were excluded. We assessed coagulation assay results and compared the results between <28 weeks (extremely preterm) and 28–32 weeks (very preterm) GA groups.

**Results:**

Coagulation assays were obtained at birth in 144 infants (144/2577; 5.5%) of whom 104 fulfilled the inclusion criteria. We found similar median PT and APTT values for extremely and very preterm infants (PT: 18.1 vs. 18.7 s [*p*‐value = 0.400]; APTT: 44.2 vs. 47.7 s [*p*‐value = 0.252], respectively).

**Conclusion:**

We found similar coagulation assay results at birth for extremely and very preterm infants; however, results deviated considerably from some of the published reference ranges. This may be due to differences between analysers and reagents, underlining the need for reference ranges calibrated to the equipment used per NICU.

**Highlights**

- Routine coagulation assessment at birth remains a common practice in some European neonatal intensive care units.
- We found that the coagulation assay results at birth deviated considerably from published reference ranges, and the commonly used distinction between extremely and very preterm infants for setting reference values is not supported by our results.
- This study highlights the complexity in the interpretation of coagulation screen results in preterm infants and emphasizes the need for caution in making treatment decisions based on abnormal coagulation screens results alone.

## INTRODUCTION

Preterm infants have a different haemostatic system compared with adults and children, with decreased levels of coagulation factors which translate into prolonged coagulation assay results. Since preterm infants also have a higher bleeding incidence, neonatologists sometimes administer fresh‐frozen plasma (FFP) to non‐bleeding infants with prolonged coagulation assay results to correct these, in the hope of reducing bleeding risks. However, available evidence for reference ranges of coagulation assays (prothrombin time [PT] and activated partial thromboplastin time [APTT]) in preterm infants is limited [1]. Importantly, laboratories use different analysers and reagents, each with different compositions and varying sensitivity to factors like C‐reactive protein (CRP) and coagulation factor deficiencies, which also limits the generalizability of the reference ranges published in the literature [2]. Prospective validation of these analysers against published reference values is difficult, as coagulation testing at birth is usually not part of routine care and additional blood sampling is unwarranted because it might contribute to iatrogenic anaemia. Moreover, further distinctions are often made in the literature for reference values based on gestational age (GA) among those born preterm; however, the basis for this distinction is anything but robust and may not be required [3, 4, 5]. We therefore set out to evaluate coagulation assay results on the day 1 of life in preterm infants admitted to the neonatal intensive care unit (NICU).

## MATERIALS AND METHODS

### Study population

We conducted a single‐centre retrospective study of coagulation assay results (PT and APTT) at birth in extremely and very preterm infants admitted to the NICU at the Leiden University Medical Center (LUMC), one of nine NICUs in the Netherlands. All preterm infants with a GA < 32 weeks admitted between 1 January 2004 and 1 January 2020 in whom coagulation assays were obtained at birth (defined as within the first 24 h of life) were included. We excluded infants diagnosed with major intraventricular haemorrhage (IVH ≥ Grade III according to Papile et al. [6]) during the first 24 h of life, infants who received FFP transfusion prior to the coagulation assay and infants in whom no cranial ultrasound was performed. All infants received 1 mg vitamin K intravenously at birth as part of routine care. The Medical Ethics Committee of the hospital reviewed our application for the re‐use of patient data for research and waived the need for informed consent (G17.045).

### Coagulation assays

Coagulation testing at birth was not part of standard care during the study period and was performed only at the discretion of the attending physician, guided by the clinical presentation of the infant. Peripheral blood samples were collected in citrated tubes, centrifuged at 3000 RCF for 8 min at room temperature and subsequently analysed. The APTT assays were performed using STA Cephascreen reagent on the STA‐Rack analyser from 2004 to 2007, the STA‐R (Evolution) analyser from 2007 to 2017 and the STA‐R Max analyser from 2017 to present (STA series: Diagnostica Stago, Asnières‐sur‐Seine, France). The PT assays were performed on the Electra analyser from 2004 to 2010 using the Recombiplastine reagent (Instrumentation Laboratory/Werfen, Barcelona, Spain), Neoplastine‐R reagent was used on the STA‐R (Evolution) analyser from 2010 to 2017 and the STA‐R Max analyser from 2017 to present (STA series: Diagnostica Stago, Asnières‐sur‐Seine, France). Where reagents and/or analysers have been changed over the years, an alternative with equivalent sensitivity was sought wherever possible. All coagulation assays were performed in accordance with instructions from the respective manufacturers. Coagulation assay results above the measuring range were reviewed by an expert panel (J.E., J.v.d.B. and E.L.) to evaluate whether they should be classified as artefacts.

### Local transfusion guideline

The NICU guideline for plasma transfusions remained unchanged up to 2019, using the reference values from Andrew et al. for all preterm infants in the absence of ranges validated for extreme prematurity [7]. At the time, the guideline stipulated that non‐bleeding infants with severe coagulopathy (defined as a coagulation assay result more than twice the average from the ranges as presented by Andrew et al.) should be treated with FFP transfusion (10 mL/kg over 30–60 min) and an additional dose of vitamin K [8]. At the end of 2019, the local guideline was revised to its present version, incorporating the reference values by Neary et al. for infants born below 28 weeks gestation [5]. Furthermore, the use of plasma in infants with abnormal coagulation values but without clinical bleeding is no longer indicated. This is currently indicated only for infants with clinically evident bleeding (non‐IVH) or bleeding due to congenital/hereditary coagulation factor deficiencies, and for infants with (suspicion of) disseminated intravascular coagulation.

### Outcome measures

In this study, we evaluated the PT and APTT test results at birth, stratified by GA at birth: below 28 weeks (defined as extremely preterm infants) and between 28 and <32 weeks (defined as very preterm infants). We reported the percentage of infants in whom coagulation assays were performed and compared the ranges between the GA groups. The following characteristics were collected: sex, GA at birth, birth weight, small for GA (defined as birth weight <10th percentile), multiple birth and delivery mode. All study variables were collected from electronic health records.

### Statistical analysis

All computations were performed using STATA Statistical Software version 16.1 (Texas, USA). Baseline variables following a normal distribution were presented as means (standard deviation) and non‐normally distributed values as medians (interquartile range [IQR]). Coagulation ranges were presented separately for extremely and very preterm infants using the 5th–95th percentile. The Mann–Whitney *U* test was used to compare the coagulation assay results between the GA groups. To assess changes in the different analysers and reagents used over the years, PT and APTT values per analyser period were presented.

## RESULTS

During the study period, coagulation assays at birth were performed in 144 of 2577 (5.5%) preterm infants. Following application of the exclusion criteria and assessment of possible artefacts, the remaining 104 infants were included in the analysis (Figure S1). Baseline characteristics for the entire study cohort and the infants included in the analysis are shown in Table 1. The study population included proportionally more male infants than the baseline population, and with a slightly lower GA.

**TABLE 1:** Patient characteristics.

	All infants included in the analysis (*n* = 104)	All infants (*n* = 2577)
Female sex, *n* (%)	38 (37)	1205 (47)
GA at birth (weeks), median (IQR)	28 (27–30)	29 (28–31)
Birth weight (g), median (IQR)	1232 (1020–1491)	1252 (980–1520)
Small for gestational age, *n* (%)	5 (5)	224 (9)
Multiple birth, *n* (%)	28 (27)	1013 (39)
Caesarean section, *n* (%)	48 (46)	1253 (49)
Mortality during NICU admission, *n* (%)	21 (20)	169 (7)

Abbreviations: GA, gestational age; IQR, interquartile range; NICU, neonatal intensive care unit.

**FIGURE 1:**
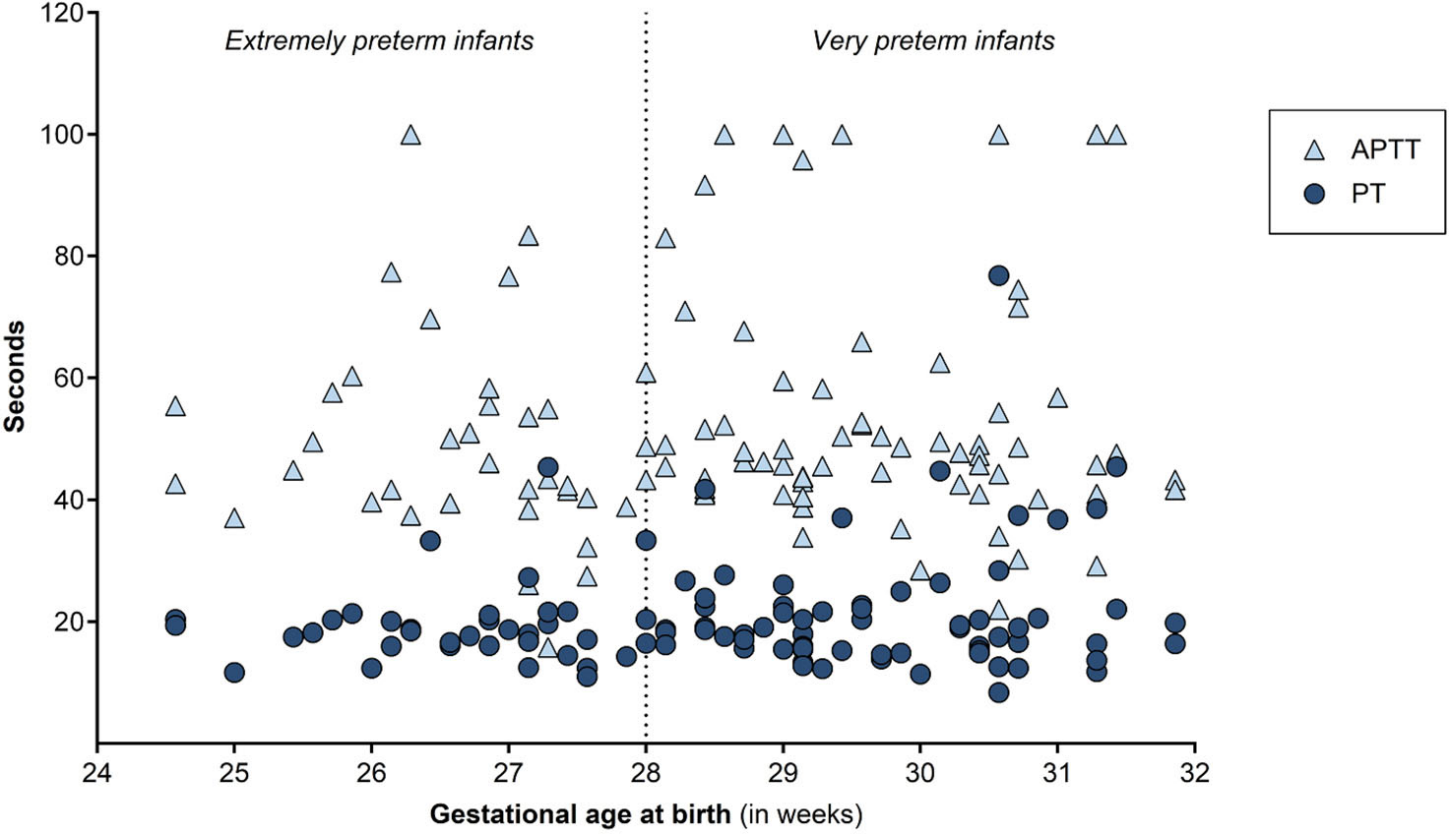
Coagulation assay results at birth. APTT, activated partial thromboplastin time; PT, prothrombin time.

The coagulation assay results for extremely and very preterm infants are presented in Figure 1. The median PT values were similar for extremely and very preterm infants (18.1 vs. 18.7 s; *p* = 0.40) with comparable 5th–95th percentile ranges (11.7–33.3 s vs. 12.3–41.8 s). Median APTTs at birth were also similar in extremely preterm and very preterm infants (44.2 vs. 47.7 s; *p* = 0.25), with 5th–95th percentile ranges 26.1–83.4 and 30.3–100.0 s, respectively. PT and APTT values per analyser period are presented in Tables 2a and 2b.

**TABLE 2a:** PT values per analyser period.

Period	Analyser	Reagents	PT (s)^c^	Number of infants
2004–2009	Electra^a^	Recombiplastine^a^	17.0 (11.8–37.5)	56 (56/104; 53.8%)
2010–2016	STA‐R (Evolution)^b^	STA Neoplastine‐R^b^	19.6 (13.7–41.8)	42 (42/104; 40.4%)
2017–2020	STA‐R Max^b^	STA Neoplastine‐R^b^	23.1 (12.3–44.8)	6 (6/104; 5.8%)

Abbreviation: PT, prothrombin time.

^a^ Instrumentation Laboratory/Werfen, Barcelona, Spain.

^b^ Diagnostica Stago, Asnières‐sur‐Seine, France.

^c^ Median, 5th–95th percentile.

**TABLE 2b:** APTT values per analyser period.

Period	Analyser^a^	Reagents^a^	APTT (s)^b^	Number of infants
2004–2006	STA‐Rack	STA Cephascreen	44.1 (29.2–60.9)	26 (26/104; 25.0%)
2007–2016	STA‐R (Evolution)	STA Cephascreen	48.7 (28.5–100.0)	72 (72/104; 69.2%)
2017–2020	STA‐R Max	STA Cephascreen	66.8 (38.8–100.0)	6 (6/104; 5.8%)

Abbreviation: APTT, activated partial thromboplastin time.

^a^ Diagnostica Stago, Asnières‐sur‐Seine, France.

^b^ Median, 5th–95th percentile.

## DISCUSSION

Our study showed similar coagulation assay results at birth in extremely and very preterm infants and significant discrepancies with literature‐based reference ranges. This is in contrast with the local guideline, which advises using different normal ranges for the two groups.

**FIGURE 2:**
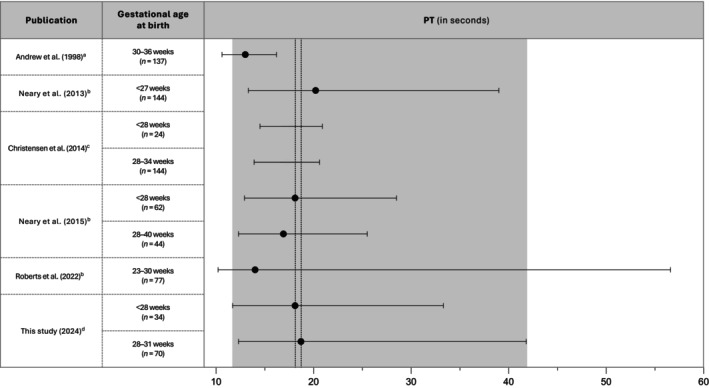
Prothrombin time (PT) ranges from literature and coagulation assay results in this study. ^a^Mean (95% confidence interval), ^b^median (min‐max), ^c^5th–95th percentile (no median reported), ^d^median (5th– 95th percentile). The dotted lines represent the median values of PT in this study.

**FIGURE 3:**
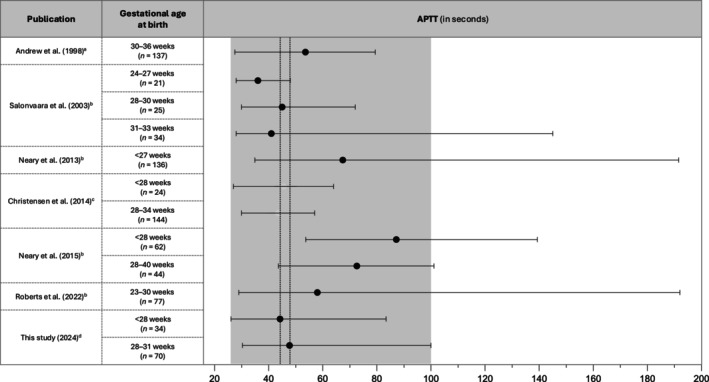
Activated partial thromboplastin time (APTT) ranges from literature and coagulation assay results in this study. ^a^Mean (95% confidence interval), ^b^median (min‐max), ^c^5th–95th percentile (no median reported), ^d^median (5th– 95th percentile). The dotted lines represent the median values of APTT in this study.

To the best of our knowledge, there are six other studies that published reference values for PT and APTT at birth in preterm infants (Figures 2 and 3 for reference values and Table S1 for analysers and reagents used in these studies) [3, 4, 5, 8, 9, 10]. In 1988, Andrew et al. published the first study on neonatal reference ranges [8], showing a median APTT of 47.7 s. However, the median PT of 18.7 s for the infants in this study was not covered by that confidence interval. Since these reference ranges formed the basis of the local guideline, we may have misclassified a proportion of infants as having a ‘prolonged’ PT, which in turn may have resulted in additional treatment with vitamin K or plasma. Andrew et al. used an assay with rabbit brain thromboplastin, whereas the laboratory currently uses recombinant thromboplastin which is known to be more sensitive to coagulation factor deficiencies [11]. Additionally, the majority of infants included by Andrew et al. were born after 32 weeks gestation. As the activity of coagulation factors gradually rises with increasing GA, this may have resulted in shorter PT times in the study by Andrew et al. [12, 13]. Similar to Andrew et al., Salonvaara et al. assessed coagulation assay results at birth in infants born <37 weeks gestation [3]. Only APTT ranges could be compared with their results as they did not publish PT ranges other than PT‐INR ranges. APTT ranges were presented separately by Salonvaara for different gestational groups, with the median APTT showing a decreasing trend with increasing GA. The median APTT values found appear similar to their results, but their applicability is limited because of different cut‐off values used for the GA.

The studies by Christensen et al. and Roberts et al. both established reference intervals for coagulation assays using umbilical cord blood draws [4, 10]. The article by Christensen unfortunately did not report the corresponding median values, which limits translation to our findings. However, all our median values of PT and APTT fall within their ranges for equivalent gestations. Comparable to our findings, Christensen found similar ranges for extremely and very preterm infants [4]. The study by Roberts found wider ranges of normal for APTT and PT in extremely preterm infants despite their larger sample size, possibly indicating naturally occurring variation in infants with immature haemostasis [10]. Our median APTT and PT for extremely preterm infants are within the presented ranges. However, one can question whether cord‐blood‐derived reference ranges can be readily compared with values from peripheral blood draws in delivered preterm infants, given the physiological changes and routine vitamin K administration after birth.

Neary et al. published two articles on reference ranges for extremely preterm infants based on peripheral blood draws, which presented similar reference ranges for PT values at birth in both studies. However, they found a considerably shorter median APTT in their first study in 2013 compared with their second study in 2015 (67.4 vs. 87.2 s), again highlighting the variability in coagulation assay results when using different analysers and reagents [5, 9]. The median PT in our study population for this GA was equivalent to the PT published by Neary in 2015. However, we found a median APTT of 44.2 s for the extremely preterm infants, which falls outside the range found by Neary et al. [5]. Since our current guideline incorporates the ranges from 2015 for coagulation assays in extremely preterm infants, this might have led us to wrongly classify a proportion of extremely preterm infants as having APTT values ‘within the reference range’. Importantly, APTT is a non‐standardized and non‐harmonized test, as APTT reagents differ in their composition and hence average coagulation assay results (among others due to differences in activators used in the reagents) and sensitivity to coagulation factor deficiencies, CRP and unfractionated heparin. These differences may be of greater influence in APTT testing in preterm infants, given that the activity of vitamin K‐dependent factors is further reduced [12]. Differences among PT reagents are smaller, yet the PT also is a non‐standardized test, as reagent composition (including the thromboplastin source), method and analyser influence the PT results. The international normalized ratio (INR) for PT has resulted in improved harmonization and interpretation of PT results for patients using vitamin K antagonists but does not completely solve the issue of inter‐laboratory differences in PT (INR) results [14].

When evaluating the PT and APTT values on the different analysers used in our centre throughout the years, we found that the median values in the last period were longer compared to the two previous periods. This could be due to the very small number of infants in whom coagulation assays were performed during this period, which could have resulted in differences by chance. Alternatively, it could reflect a more conscious practice of clinicians to only request coagulation testing in sicker infants in the latter years.

Besides the lack of validated reference ranges specific to the different analysers and reagents, there are other concerns about the clinical use of coagulation assays in preterm infants. To date, it is still unclear whether prolonged coagulation values are an indicator of increased bleeding risk. Only observational evidence is available in answer to this question, reporting contrasting findings on the association between prolonged coagulation assays and bleeding risk in infants. It has been suggested that preterm infants maintain an equilibrated haemostatic system, even with reduced coagulation factor levels translating into longer coagulation values, through effective counteraction of other factors promoting haemostatis such as increased haematocrit and mean corpuscular volume and low levels of anticoagulant factors such as proteins C and S [15]. Furthermore, it is not clear whether FFP transfusions can substantially correct abnormal coagulation values and thereby reduce the risk of bleeding [16, 17]. Only one randomized trial was conducted more than 20 years ago to assess the potential beneficial effect of prophylactic FFP, which showed no improvement in terms of severe morbidity and/or mortality among infants [18]. The neonatal haemostatic system may not respond to treatment interventions as conventionally thought, although the use of FFP in adults also remains controversial [19, 20, 21, 22]. The recent PlaNeT‐2/MATISSE trial demonstrated that, contrary to general assumptions, a liberal platelet transfusion threshold resulted in higher mortality and/or bleeding rates compared with a restrictive threshold [23]. In the context of a potential treatment with limited supporting evidence of its effectiveness, the question arises: what is the benefit of routinely assessing these coagulation values at birth?

Additionally, coagulation assays require a relatively large blood sample (1.3 mL in our analyser), which is considerably more than the volume required for many other tests. Preterm infants have a very small circulating blood volume, equivalent to approximately 70 mL for a 1000‐g infant, and are known to lose a substantial portion of this volume during admission due to frequent laboratory testing [24, 25]. It is important to be wary of iatrogenic anaemia resulting from phlebotomy losses, and clinicians should consider this when deciding to perform coagulation assays in preterm infants.

Currently, in the NICU guideline, it is not indicated to administer FFP for prolonged coagulation assay results in non‐bleeding infants. However, this was more prevalent in our NICU a few years ago, with more than half of plasma transfusions being given for coagulopathy without active bleeding [26]. According to a recent survey conducted across Europe, not all NICUs have adapted to more restrictive handling. To date, 11% of European centres continue to routinely assess coagulation values and 39% opt for FFP transfusions solely based on extended coagulation assays, even in non‐bleeding infants [27].

Over the last years, the use of thromboelastography has been proposed as an alternative to standard coagulation assays. Thromboelastography assesses the different phases of the coagulation process and requires a smaller volume of blood, but to date there is no evidence that possible treatment based on abnormal thromboelastography test results can effectively prevent bleeding in preterm infants [28, 29].

This study has limitations. Since determining coagulation at birth was not part of standard care, it probably led to a selection of infants with a clinically sicker presentation that prompted the attending physician to order a coagulation assay. This limits the generalizability of our results to the general preterm population, although we do believe that in this sicker group of infants it is of paramount importance to interpret coagulation assay results correctly and to avoid prophylactic administration of FFP in non‐bleeding neonates. Furthermore, in this article we only assessed coagulation assay results specific for the analysers and reagents used in our laboratory, which limits the generalizability to other hospitals. However, with this article, we aim to raise awareness for the complexity and the inter‐variability between different haemostasis analysers and reagents. Additionally, we encourage others to critically assess the characteristics and performance of their own analysers, for example, by retrospectively comparing coagulation results with locally applied reference values.

We conclude that, in the absence of reference values calibrated with the analysers and reagents used per NICU, neonatologists should be cautious about interpretation and possible treatment decisions based on supposedly ‘prolonged’ coagulation values. The commonly used distinction between extremely and very preterm infants for setting reference values is not corroborated by our results.

## CONFLICT OF INTEREST STATEMENT

The authors declare no conflicts of interest.

## Supporting information


**Figure S1.** Flowchart.
**Table S1**. Coagulation ranges from the literature and this study, including analysers and reagents used.

## Data Availability

Data from this study are available on reasonable request to the corresponding author, with prior written consent.
