# Supplementary material for: Coagulation assay results at birth in preterm infants: A cohort study highlighting the relevance of local reference values for interpretation
Source: Vox Sang. 2024 Nov 18;120(1):55–62. doi: 10.1111/vox.13766 (PMC11753824; doi:10.1111/vox.13766)
Supplement: Supplementary file 1 — Figure S1. Flowchart. Table S1. Coagulation ranges from the literature and this study, including analysers and reagents used. [file VOX-120-55-s001.pdf]

Supplementary Materials

Figure S1. Flowchart

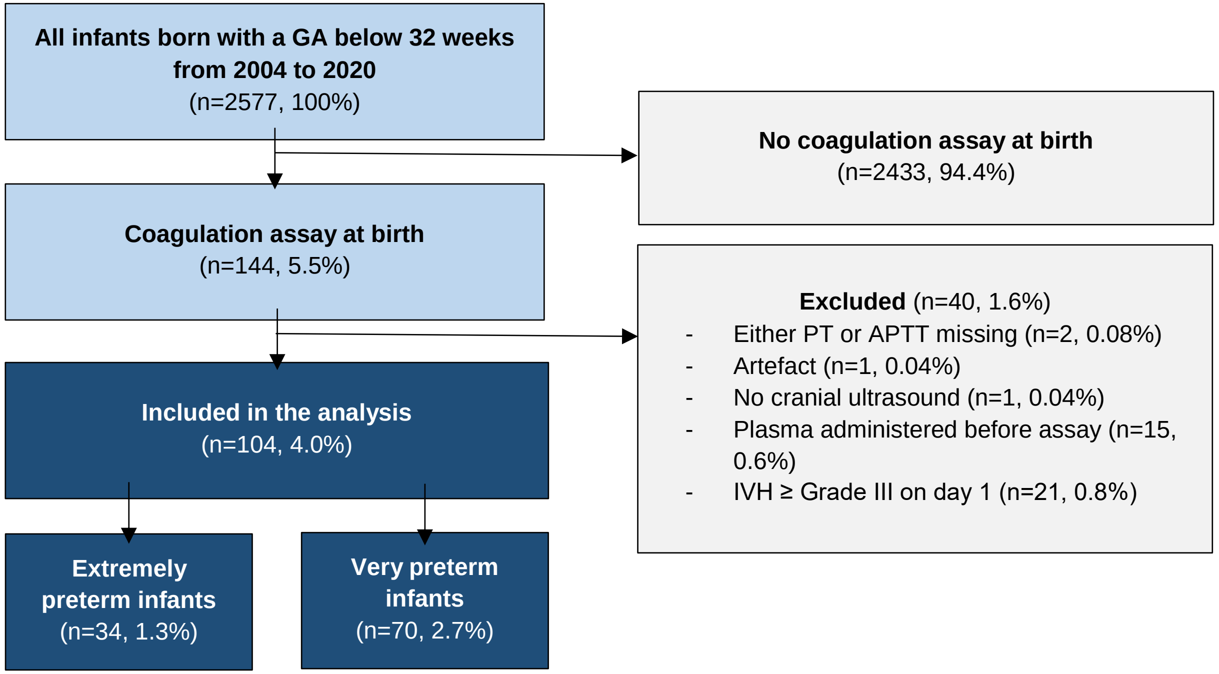

Table S1. Coagulation ranges from literature and this study, including analysers and reagents used

Publication	Study population	Blood samples	Analyser	PT Reagents	APTT Reagents	PT, in seconds	APTT, in seconds
**Andrew et al.** (1988)	Preterm infants born at 30 to 36 weeks gestation (n=137)	2 mL blood samples obtained via venepuncture blood draw within first day of life and collected in tubes containing buffered sodium citrate	*Not reported*	Thromboplastin C Plus (Dade, Marburg, Germany)	Actin FS (Dade, Marburg, Germany)	**13.0 (10.6 – 16.2)***	**53.6 (27.5 – 79.4)***
**Salonvaara et al.** (2003)	Preterm infants born below 37 weeks gestation (n=125)	1.8 mL blood samples obtained from peripheral arterial or venous catheters within two hours after birth and collected in citrated tubes	Thrombolyzer (Behnk Elektronik, Norderstedt, Germany)	*Not applicable*	Cephotest (Nycomed Pharma, Oslo, Norway)	*Only reported PT expressed as INR*	GA 24-27 weeks at birth (n=21)): **36 (28 – 48)**** GA 28-30 weeks at birth (n=25)): **45 (30 – 72)**** GA 31-33 weeks at birth (n=34)): **41 (28 – 145)****
**Neary et al.** (2013)	Preterm infants born below 27 weeks gestation (n=183)	1.3 mL blood samples obtained via peripheral venous cannula or umbilical arterial or venous catheters within hours after birth and collected in citrated tubes	ACL 9000 (Instrumentation Laboratory, Bedford, USA) / ACL TOP (Instrumentation Laboratory, Bedford, USA)	PT-S reagent (Instrumentation Laboratory, Bedford, USA) / Recombinant PlasTin 2G (Instrumentation Laboratory, Bedford, USA)	APTT-SP (Instrumentation Laboratory, Bedford, USA) / SytnASil (Brennan & Co, Dublin, Ireland)	**20.2 (13.3 – 39)**** (n=144)	**67.4 (34.9 – 191.6)**** (n=136)

**Christensen et al.** (2014)	Preterm infants born below 35 weeks gestation (n=168)	Cord blood samples obtained from the umbilical vein after birth	STA compact (Diagnostica Stago, Asnières-sur-Seine, France)	STA-Neoplastine CI Plus (Diagnostica Stago, Asnières-sur-Seine, France)	STA PTT Automate (Diagnostica Stago, Asnières-sur-Seine, France)	GA <28 weeks at birth (n=24): **14.5 – 20.9*****<br><br>GA 28-34 weeks at birth (n=144): **13.9 – 20.6*****	GA <28 weeks at birth (n=24): **27 – 64*****<br><br>GA 28-34 weeks at birth (n=144): **30 – 57*****
**Neary et al.** (2015)	Preterm infants born below 30 weeks gestation (n=116)	Blood samples obtained via peripheral non-heparinized lines on admission at the NICU and collected in citrated tubes	ACL TOP coagulometer (Beckman Coulter Inc., Galway, Ireland)	HemosIL RecombiPlasTin (Instrumentation Laboratory, Bedford, USA)	HemosIL APTT Lyophilized Silica (Instrumentation Laboratory, Bedford, USA)	GA <28/40 weeks at birth (n=62): **18.1 (12.9 – 28.5)****<br><br>GA >28/40 weeks at birth (n=44): **16.9 (12.3 – 25.5)****	GA <28/40 weeks at birth (n=62): **87.2 (53.7 – 139.3)****<br><br>GA >28/40 weeks at birth (n=44): **72.6 (43.6 – 101.1)****
**Roberts et al.** (2022)	Preterm infants born at 23 to 30 weeks gestation (n=120)	3 mL cord blood samples obtained from the umbilical vein after birth and collected in citrated tubes	Behring Coagulation Timer (Siemens Healthineers, Erlangen, Germany)	*Not reported*	*Not reported*	**14 (10.2 – 56.6)**** (n=79)	**58 (29-192)**** (n=77)
**This study** (2024)	Preterm infants born below 32 weeks gestation (n=104)	1.3 mL peripheral blood draws were collected in citrated tubes	Electra analyser (Instrumentation Laboratory/Werfen, Barcelona, Spain) / STA-Rack analyser, STA-R (Evolution), STA-R Max analyser, (Diagnostica Stago, Asnières-sur-Seine, France)	Recombiplastine reagent (Instrumentation Laboratory/Werfen, Barcelona, Spain) / STA Neoplastine-R (Diagnostica Stago, Asnières-sur-Seine, France)	STA Cephascreen (Diagnostica Stago, Asnières-sur-Seine, France)	GA<28 weeks at birth (n=34): **18.1 (11.7 – 33.3)******<br><br>GA 28 to <32 weeks at birth (n=70): **18.7 (12.3 – 41.8)******	GA<28 weeks at birth (n=34): **44.2 (26.1 – 83.4)******<br><br>GA 28 to <32 weeks at birth (n=70): **47.7 (30.3 – 100)******

**Mean (95% confidence interval), **median (min-max), ***5th to 95th percentile (no median reported), ****median (5th to 95th percentile).*
